# Characteristics, Prognosis and Reasons for Opting-Out of Treatment in Patients with Untreated Pancreatic Cancer

**DOI:** 10.3390/curroncol33020116

**Published:** 2026-02-16

**Authors:** Morten Ladekarl, Mogens Tornby Stender

**Affiliations:** 1 Department of Oncology, Clinical Cancer Research Center, Aalborg University Hospital, 9000 Aalborg, Denmark; 2 Department of Clinical Medicine, Aalborg University, 9260 Gistrup, Denmark; 3 Department of Surgery, Aalborg University Hospital, 9260 Gistrup, Denmark

**Keywords:** pancreas cancer, register study, opting out of treatment, disparity of treatment, frailty, prognosis

## Abstract

*Background:* About 40% of patients with pancreatic cancer (PC) are left untreated. Identification of the modifiable factors for opting out could increase the number eligible for treatment. *Methods:* We first assessed the completeness of registration. Next, we identified patients residing in the North Denmark Region, included 2023/24 in the Danish Pancreas Cancer Database (DPCD), registered as “no treatment”. We supplemented register data with health record data, including reasons for opting out of treatment. *Results:* Registration in DPCD was complete compared to the National Clinical Cancer Database, except for one patient. Six patients had other tumors. Of a total of 91 patients, 79% were >75 years old, 2/3 were in performance status (PS) > 2, more than half were socially or physically fragile, while 42% had significant comorbidity. Only 20% were referred to an oncologist. The median overall survival was 2 months, and the 1-year survival was 6%. Clinical stage and PS were prognostic in multivariable analysis. In 70%, poor PS was a reason for opting out of treatment, while 11% declined treatment without objective reasons. *Conclusions:* Poor PS, frailty, or patients’ wishes explained 89% opting out of treatment. On a patient level, modifiable factors seem limited in this population.

## Simple Summary

This study aimed to assess the characteristics and reasons for opting out of treatment in the ~40% of patients with pancreatic cancer who are left untreated. We first assessed the completeness of registration and then obtained clinical data regarding patients residing in the North Denmark Region, included 2023/24 in the Danish Pancreas Cancer Database, registered as “no treatment”. Registration was 99% complete compared to the National Clinical Cancer Database. Of 91 patients, 79% were >75 years old, 2/3 were in poor performance status (PS), more than half were socially or physically fragile, while 42% had significant comorbidity. Only 20% were referred to an oncologist. The median overall survival was 2 months, and the 1-year survival was 6%. Clinical stage and PS were prognostic. Poor PS, frailty, or patients’ wishes explained 89% opting out of treatment, and 11% declined treatment without objective reasons. On a patient level, modifiable factors seem limited in this population.

## 1. Introduction

Pancreatic cancer (PC) represents a prominent and rising cause of cancer-related morbidity and mortality, especially in high-income regions [1]. Surgery combined with systemic chemotherapy provides the best chance of long-term survival, but the diagnosis is often late and associated with high disease burden, old age and/or frailty [2,3], reducing patients’ eligibility for treatment [4,5]. Population-based studies have shown that less than 20% of patients newly diagnosed with PC are treated by surgery (median overall survival (mOS) 16–27 months), while approximately 40% have palliative chemotherapy as their first treatment (mOS 6–9 months) [6,7,8].

A significant number of patients are left untreated, in the sense that they receive no surgery or antineoplastic treatment [4,9]. In 3852 fully insured patients with PC included from 2010 to 2020 in the Kaiser Permanente Northern California Cancer Registry, 22% with non-metastatic and 44% with metastatic disease never received cancer-directed treatment [10]. In a study of 9559 patients diagnosed from 1995 to 2004 with stage I PC and included in the US National Cancer Database (NCDB), only 29% underwent surgery [4]. Similarly, of a total of 1177 PC cases registered in Denmark in 2023–2024 [11], 42% never received active treatment [12]. This untreated group of patients has a dismal prognosis (mOS of only 1.1–2.9 months) [6,7,8,13].

Data on untreated patients with PC are scarce, as many such patients are not referred to hospitals, and in most registers, the registration and data completeness (even including death [14]) can be questioned. A recent population-based US study, where patients were selected by being fully insured, showed that increasing age and, in metastatic disease, a Charlson Comorbidity Index (CCI) 3+, but not race/ethnicity or gender, correlated with no treatment [10]. In an older US study of stage I patients included in the NCDB, age > 65, Afro-American race, lower annual income, less education, no private insurance, treatment at low-volume or community centers, and tumor site in the pancreatic head were all correlated with no treatment by surgery [4].

Data from unselected populations are important to assess modifiable factors that could increase the number of patients eligible for active treatment, especially as untreated patients’ poor prognosis has a high impact on the mOS of the entire PC population [6]. In this report, we aimed to identify reasons for no treatment and to describe the characteristics of an unselected population of patients with PC, where active treatment was never started.

## 2. Materials and Methods

We first assessed the completeness of registration of PC patients in the Danish Pancreas Cancer Database (DPCD). This prospective clinical register includes patients with a diagnosis of pancreas cancer, including carcinomas of the pancreas, ampulla, and duodenum and excludes neuroendocrine tumors and rare non-carcinoma subtypes [15]. Another database, the Danish National Clinical Cancer Database (DNKK) [11] (founded in 1942 as the Danish Cancer Registry), has high completeness and includes patients with any cancer diagnosis, both assessed clinically and by histology [16]. In the present study, to assess register completeness, we identified and characterized patients residing in the North Denmark Region who, in the period 1 July 2023 to 30 June 2024, were registered with a pancreatic cancer diagnosis in the DNKK database and not in the DPCD.

Next, from the DPCD, we identified a cohort of patients residing in the North Denmark Region and included in the registry from 1 July 2023 to 30 June 2024, registered as “no treatment”. In the North Denmark Region, scans from all patients with a suspicious pancreatic lesion on CT are prospectively reevaluated at the regional multidisciplinary pancreas tumor conference involving pancreatic surgeons, oncologists, radiologists, gastroenterologists, and pathologists, and cases are diagnosed, staged according to TNM, and registered in both the DNKK and DPCD, independently of whether the patients are candidates for treatment or not. According to the Danish national guidelines, a high-quality contrast-enhanced CT scan was supplemented by magnetic resonance imaging, positron emission tomography, or endoscopic ultrasound imaging when indicated. Operable patients with borderline or non-resectable non-metastatic tumors were also evaluated at the Danish national pancreatic multidisciplinary conference to obtain consensus regarding treatment plans. For TNM-staging we used the American Joint Committee on Cancer 8th edition staging system [17], based on findings on images and supplemented with histopathological findings when available.

We retrieved demographic and clinical prospective data from the DPCD, supplemented by data retrospectively extracted from the patients’ electronic health records (EHRs). The data included patient characteristics (sex, age, weight, height, vital status, Eastern Cooperative Oncology Group performance status (PS), American Association of Anesthesiologists (ASA) score, CCI [18], use of anticoagulants, smoking status, alcohol consumption >10 units/week, previous or synchronous other cancer diagnoses), frailty indicators (use of a wheelchair and/or walker, public domestic help), tumor characteristics (primary tumor location and size, TNM-stage, histological confirmation), blood tests at baseline (cancer antigen (CA) 19-9, plasma (p)-albumin, p-bilirubin), demographics (municipality of residence, traveling distance to the treating center), socio-economic factors (number of household members, labor market status, type of housing), and use of palliative procedures (biliary tract stenting, palliative surgical procedures). Finally, we registered whether patients were referred to an oncological department and, from a journal audit, their reasons for opting out of treatment.

*Statistics:* Categorical variables are presented as the number of observations with relative frequencies, while continuous variables are presented as the mean +/− standard deviation (SD) or medians with min–max. Kaplan–Meier survival analysis was done, and survival curves were constructed according to the selected exposure variables. Cox regression analysis was used to examine the prognostic effect of the selected exposure variables, including the age, sex, tumor stage, PS and municipality of residence. The data met the assumption of “proportional hazards”. The follow-up time is calculated from the registered start of the course to either death or end of the follow-up (12 April 2025). A two-sided *p*-value of <0.05 is considered significant. Data were analyzed, and graphs were created with STATA 18.5 (Stata Corp LLC, College Station, TX, USA).

## 3. Results

### 3.1. Completeness of the DPCD Database in the North Denmark Region

A total of 162 patients residing in the North Denmark Region were registered in the DPCD database during the inclusion period. Eleven patients were registered with a PC diagnosis in the DNKK database but not in DPCD. Of these, eight had pancreatic neuroendocrine tumors (pNET), one patient with PC was not referred to the regional multidisciplinary pancreas tumor conference, while two patients with PC were already registered in the DPCD prior to the inclusion period. As only one patient with verified PC was registered in DNKK but not in DPCD, the registration in DPCD compared to DNKK was almost complete (99%).

### 3.2. Characteristics of Untreated Patients

Of the 97 patients registered in DPCD with “no treatment”, two patients had pNET, one had pancreatic lymphoma, while three patients had cancers arising outside the pancreas. These were excluded from further analyses, leaving 91 patients for further assessments.

Demographic and clinical data are given in Table 1.

This was an elderly population, as 79% of patients were more than 75 years old, and 24% were over 85. More than two-thirds of patients were in poor PS (PS > 2). The tumor burden was high with a median CA 19-9 of 898 kU/L and stage IV disease in more than half. Social and physical frailty was evident, with more than 50% either living alone, using a wheelchair or walker, or having public domestic help. Significant comorbidity (CCI of 2+) was present in 42% of patients, and more than 1/3 of patients had prior or concurrent other cancers. Only 20% were referred to the department of oncology for evaluation of treatment. Many patients deteriorated quickly during the work-up period. About half of the diagnoses were based on imaging alone. Histological verification was not associated with stage and was based on biopsies in all cases. Patients in stage IV were, on average, younger, had lower P-bilirubin and less frequently had biliary stenting, as well as larger primary tumors and much higher CA 19-9 compared to patients with non-metastatic disease.

A survival curve for the whole cohort is shown in Supplementary Figure S1. The mOS was 63 days (interquartile range (IQR), 44–77 days) from the date of diagnosis. Approximately 25% of the patients died within 1 month and 75% within 5 months. The 1-year survival was only 6%.

The survival analysis (Table 2) showed younger age, advanced clinical stage, and poor PS as significant adverse prognostic factors in univariable analysis, while age was not significant in the multivariable analysis.

**Figure 1:**
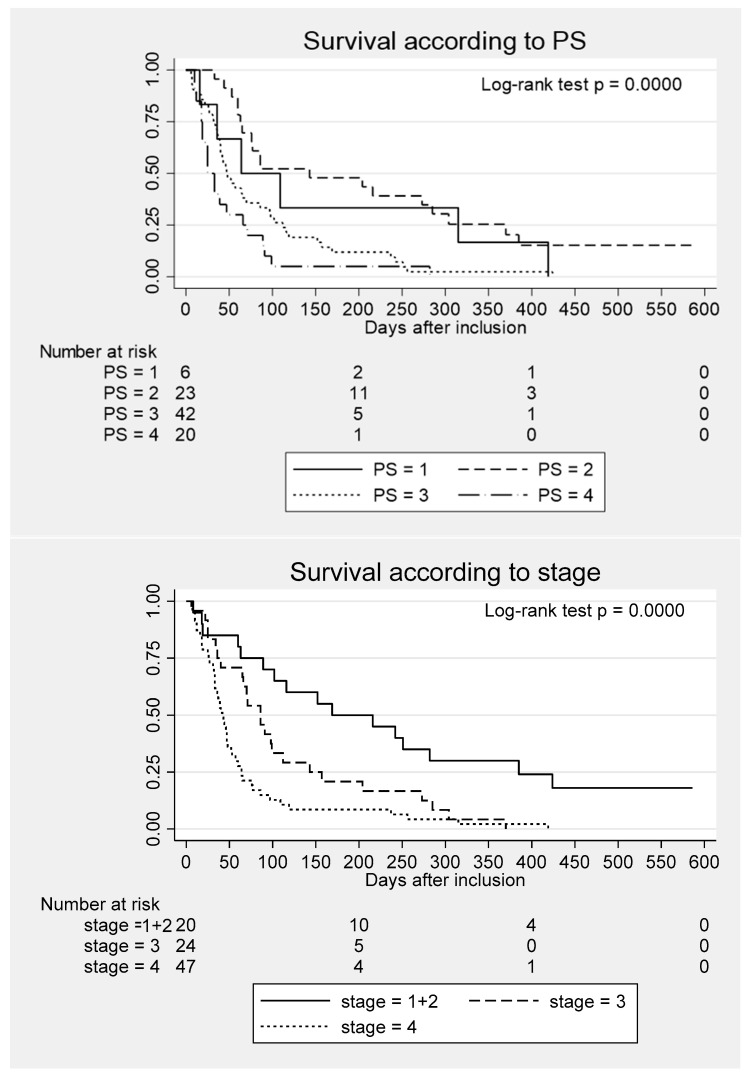
Kaplan–Meier plots of overall survival for 91 patients with untreated pancreatic cancer according to Eastern Cooperative Oncology Group performance status (PS) and clinical stage.

Figure 1 shows the overall survival according to PS and stage. Patients in PS 4 had an mOS of only 25 days (IQR, 18–66 days), and patients in stage IV had an mOS of 42 days (IQR, 33–48 days). Long-term survival was not observed for patients in stage III–IV, while survival > 1 year was achieved by six (30%) of the 20 patients in stage I or II.

### 3.3. Reasons for No Treatment

A summary of explanations for treatment opt-out is shown in Supplementary Table S1. In 70% of cases, poor performance status (PS > 2) was present as a reason for no treatment. A total of 16% declined treatment, including ten patients with no objective reasons for no treatment. In 14 patients in PS 1–2 who did not decline treatment, the reasons for no treatment were multiple, including old age, frailty, acute complications to PC, and dementia. The main reasons for opting out of treatment for patients with tumors in stage I–II were poor performance status and/or old age in 75% of cases (15 patients), while only one patient declined treatment with no objective reason.

## 4. Discussion

In this cohort, we found a high completeness of registration of PC patients in DPCD compared to DNKK, and we, therefore, believe that the study includes virtually all relevant patients in the population. A small number of patients were non-intentionally included in DPCD with neuroendocrine or misclassified tumors but could easily be identified and excluded. In a prior study comparing registered cases in DNKK and DPCD in patients from the South Region of Denmark, 6.6% of discrepant cases—mainly cases without histological diagnosis—were pancreas cancers registered in DNKK that should have been registered in DPCD [19]. This fraction was much smaller (<1%) in our cohort. Hence, the centralized reevaluation of scans from all patients with a suspicious pancreatic lesion, used in the current population, seemingly leads to very high validity and completeness of registration, including untreated cases.

Along with the disparity among oncological centers with respect to the use of combination chemotherapy [20], considerable differences have been observed in the proportion of untreated patients among the five administrative regions of Denmark. The fraction ranges from 31% to 60% with the North Denmark Region having the largest proportion of untreated patients [12]. Reasons for the regional variation are currently under investigation. One suggested cause is differences in registration completeness; however, regional variations in demographic, socio-economic, racial, and health-related factors, and access to doctors for primary and secondary care may have an impact on patients’ willingness or ability to undergo treatment [14,21]. In a US population-based register study, most patients who did not initiate treatment did so due to patient or family preference, despite being offered cancer-directed therapy by their oncologist. Other reasons for declining treatment were frailty and medical comorbidity/other malignancy in a few percent [10]. In our cohort, 89% of patients were not candidates for active treatment according to the guidelines [22], mostly due to poor PS. Only 11% of patients actively declined treatment despite no objective reasons noted for no treatment.

Surgical resection is offered to highly selected patients with tumors considered technically resectable, according to the absence of distant metastases and the extent of vascular involvement, as well as sufficient physiological capacity to tolerate major pancreatic surgery, which carries a high burden of morbidity, long-term effects and even mortality. Patients deemed to have excessive operative risk may be considered non-operable despite technically resectable disease [23,24]. In our cohort, only one patient (1%) refused surgery for early-stage cancer with no obvious reason. This partly corresponds to 4.2% (403 out of 9559 patients) who refused surgery for stage I PC in the US NCDB [4]. Further studies are needed to access whether the low number of patients referred to oncologists (only 20% in the present cohort) or other factors may correlate with patients declining active treatment, as the considerations for and against treatment depend on an often complex assessment of general condition, functional status, comorbidity, nutritional status, psychological status, medication and network/social support [5,14,25].

Our study shows that, in untreated patients, the stage of disease also has a significant impact on survival. Of note, about 30% of patients in stage I–II survived more than one year despite no treatment. Adding to this, a recent target trial emulation study suggested that 1-year survival is 50% with palliative chemotherapy compared to 77% after surgery in patients with resectable disease [26]. Therefore, chemotherapy may be considered in patients refusing surgery, and local tumor ablation—including irradiation—may also be beneficial [27]. Hence, reasonably fit patients refusing surgery, but not any treatment, should be referred to oncologists for discussion of alternatives.

The small cohort of the present study limits the statistics, and larger epidemiological studies are warranted. Among these, elucidating the reasons for non-referral to oncologists or opting out of treatment after oncological consultation could disclose modifiable factors. We did not register causes of death that may include death from comorbidities and non-natural causes in this population [28]. The BMI was recorded, but we lacked information on prior weight-loss that may be a more important and potentially modifiable parameter. An unknown number of PC cases in severely ill or very old patients are undoubtedly never diagnosed [29,30], and this number may vary in different populations. However, we found an almost complete registration of diagnosed cases, and follow-up was complete according to vital status. We prospectively registered multiple variables, and all scans were centrally reevaluated and staged according to TNM; however, reasons for opting out of treatment were assessed retrospectively by journal audit. Further validation of findings of reasons for opting out of treatment would require interviews of patients, families or treating physicians [31], but was not feasible in the current cohort.

## 5. Conclusions

In conclusion, the mOS of untreated patients with PC is only 2 months; however, it depends on the TNM-stage and PS. Patients were mostly old, and a high number were physically and/or socially fragile, explaining 89% of cases opting out of treatment; however, only 20% of patients were referred to an oncologist for specialized assessment. A total of 11% of patients refused treatment despite no objective reasons noted for no treatment. On an individual patient level, modifiable factors with an impact on eligibility for treatment seem very limited in this population.

## Figures and Tables

**Table 1:** Characteristics of 91 patients with untreated pancreatic cancer according to clinical stage.

	All Clinical Stages *n* = 91	Stage I–II *n* = 20	Stage III *n* = 24	Stage IV *n* = 47	*p*-Value
Age in years, mean (±SD)	78.1 (±9.3)	82.3 (±6.5)	81.4 (±6.6)	74.7 (±10.3)	*p* = 0.001
Sex, *n* (%); male/female	50 (55)/41 (45)	9 (45)/11 (55)	10 (42)/14 (58)	22 (47)/25 (53)	*p* = 0.96
BMI, mean (±SD)	23.8 (±4.9)	23.0 (±4.6)	23.6 (±5.0)	24.6 (±4.9)	*p* = 0.45
ECOG PS, *n* (%)					*p* = 0.052
1	6 (7)	0 (0)	0 (0)	6 (13)	
2	23 (25)	8 (40)	9 (38)	6 (13)	
3	42 (46)	9 (45)	9 (38)	24 (51)	
4	20 (22)	3 (15)	6 (25)	11 (23)	
ASA score, *n* (%)					*p* = 0.49
2	7 (7)	2 (10)	2 (8)	3 (6)	
3	58 (64)	13 (65)	18 (75)	27 (57)	
4	26 (29)	5 (25)	4 (17)	17 (36)	
CCI, *n* (%)					*p* = 0.13
0	37 (41)	12 (60)	7 (29)	18 (38)	
1	16 (17)	4 (20)	3 (13)	9 (19)	
≥2	38 (42)	4 (20)	14 (58)	20 (43)	
Smoking, *n* (%)	18 (20)	3 (15)	5 (21)	10 (21)	*p* = 0.78
Alcohol units > 10 weekly, *n* (%)	13 (14)	3 (15)	2 (8)	8 (17)	*p* = 0.67
Traveling distance *, *n* (%); 0–49 km/50+ km	50 (55)/41 (45)	10 (50)/10 (50)	11 (46)/13 (54)	29 (62)/18 (38)	*p* = 0.41
Municipality, *n* (%); city/rural	32 (35)/59 (65)	6 (30)/14 (70)	8 (33)/16 (67)	18 (38) /29 (62)	*p* = 0.84
Number of household members, *n* (%); 1/2+	49 (54)/42 (46)	13 (65)/7 (35)	15 (63)/9 (34)	21 (45) /26 (55)	*p* = 0.61
Type of housing, *n* (%)					*p* = 0.62
House	56 (61)	14 (70)	13 (54)	29 (62)	
Flat	27 (30)	6 (30)	9 (38)	12 (26)	
MD	8 (9)	0 (0)	2 (8)	6 (13)	
Employed, *n* (%)	9 (10)	0 (0)	2 (8)	7 (15)	*p* = 0.21
Public domestic help, *n* (%); yes/MD	37 (41)/1 (1)	12 (60)/0 (0)	9 (38)/0 (0)	16 (35)/1 (2)	*p* = 0.16
Use of wheelchair or walker, *n* (%)	49 (54)	12 (60)	13 (54)	24 (51)	*p* = 0.82
Referred to oncologist, *n* (%)	18 (20)	1 (5)	1 (4)	16 (34)	*p* = 0.002
Former or concurrent other cancer, *n* (%)	31 (34)	8 (40)	7 (29)	16 (34)	*p* = 0.77
Anticoagulant use, *n* (%)	44 (48)	7 (35)	11 (46)	26 (55)	*p* = 0.31
Albumin in g/L, mean (±SD)	30.7 (±6.0)	29.1 (±6.0)	32.1 (±6.2)	30.7 (±5.9)	*p* = 0.26
Bilirubin in µmol/L, median (min–max)	31 (3–566)	120 (3–481)	65 (4–566)	19 (4–251)	*p* = 0.02
CA 19-9 in kU/L, median (min–max)	898 (2 → 100,000)	242 (2–7,349)	180 (7–16,405)	6981 (4 → 100,000)	*p* < 0.001
Primary tumor, *n* (%)					*p* = 0.18
Pancreas	85 (93)	1 (5)	1 (4)	2 (4)	
Duodenum	4 (5)	17 (85)	23 (96)	45 (96)	
Papilla	2 (2)	2 (10)	0 (0)	0 (0)	
Primary tumor size in mm, mean (±SD)	39.6 (18.6)	34.3 (24.8)	35.0 (13.0)	44.2 (17.0)	*p* = 0.046
Histological verification, *n* (%)	47 (52)	9 (45)	13 (54)	25 (53)	*p* = 0.85
Bile duct stenting, *n* (%)	37 (41)	11 (55)	15 (63)	11 (23)	*p* = 0.002
Palliative surgery, *n* (%)	3 (3)	1 (5)	0 (0)	2 (4)	*p* = 0.60

Abbreviations: ASA, American Society of Anesthesiologists; BMI, body mass index; CCI, Charlson Comorbidity Index; ECOG PS, Eastern Cooperative Oncology Group performance status; MD, missing data; SD, standard deviation. * From home address to treating hospital unit. + Assessed prospectively by centralized reassessment of CT scans.

**Table 2:** Prognostic factors according to overall survival in 91 patients with untreated pancreatic cancer.

			Univariable Analysis		Multivariable Analysis	
Variable	Stratum	*n*	HR (95%CI)	*p*-Value	HR (95%CI)	*p*-Value
Age in years	<65	9	1.00	-	1.00	-
	65–74	10	0.59 (0.24–1.45)	0.25	0.46 (0.36–2.45)	0.90
	75–84	50	0.35 (0.17–0.73)	0.005	0.63 (0.29–1.36)	0.24
	85+	22	0.44 (0.20–0.96)	0.04	0.77 (0.33–1.81)	0.56
Sex	Female	41	1.00	-	1.00	
	Male	50	1.05 (0.69–1.60)	0.84	1.07 (0.68–1.67)	0.78
Clinical stage	I + II	20	1.00	-	1.00	-
	III	24	2.20 (1.14–4.27)	0.02	2.43 (1.21–4.92)	0.01
	IV	47	3.94 (2.15–7.21)	<0.001	4.90 (2.39–10.0)	<0.001
ECOG PS	1	6	1.00	-	1.00	-
	2	23	0.69 (0.27–1.73)	0.43	2.07 (0.71–6.05)	0.18
	3	42	1.83 (0.76–4.39)	0.18	4.20 (1.57–11.3)	0.004
	4	20	3.00 (1.17–7.71)	0.02	9.40 (3.01–29.3)	<0.001
Municipality	City	32	1.00	-	1.00	-
	Rural	59	0.93 (0.60–1.46)	0.76	0.96 (0.58–1.57)	0.86

Abbreviations: CI, confidence interval; ECOG PS, Eastern Cooperative Oncology Group performance status; HR, hazard ratio.

## Data Availability

The dataset is anonymized and stored as a .dta file on a secure server at Aalborg University Hospital. Individual data are unavailable due to legal restrictions.

## Supplementary Materials

The following supporting information can be downloaded at https://www.mdpi.com/article/10.3390/curroncol33020116/s1, Figure S1: Kaplan–Meier plot of overall survival for 91 patients with untreated pancreatic cancer; Table S1: Reasons for opting out treatment in 91 patients with pancreatic cancer.

## Abbreviations

The following abbreviations are used in this manuscript: ASA, American Society of Anesthesiologists; BMI, body mass index; CA, cancer antigen; CCI, Charlson Comorbidity Index; DNKK, Danish National Clinical Cancer Database; DPCD, Danish Pancreas Cancer Database; EHR, electronic health record; HR, hazard ratio; IQR, interquartile range; MD, missing data; mOS, median overall survival; NCDB, National Cancer Database; PC, pancreatic cancer; p, plasma; pNET, pancreatic neuroendocrine tumor; PS, Eastern Cooperative Oncology Group performance status; SD, standard deviation.
