# Supplementary material for: Characteristics, Prognosis and Reasons for Opting-Out of Treatment in Patients with Untreated Pancreatic Cancer
Source: Curr Oncol. 2026 Feb 16;33(2):116. doi: 10.3390/curroncol33020116 (PMC12939013; doi:10.3390/curroncol33020116)
Supplement: Supplementary file 1 [file curroncol-33-00116-s001.zip › curroncol-4105575-supplementary.pdf]

Supplementary figures and tables

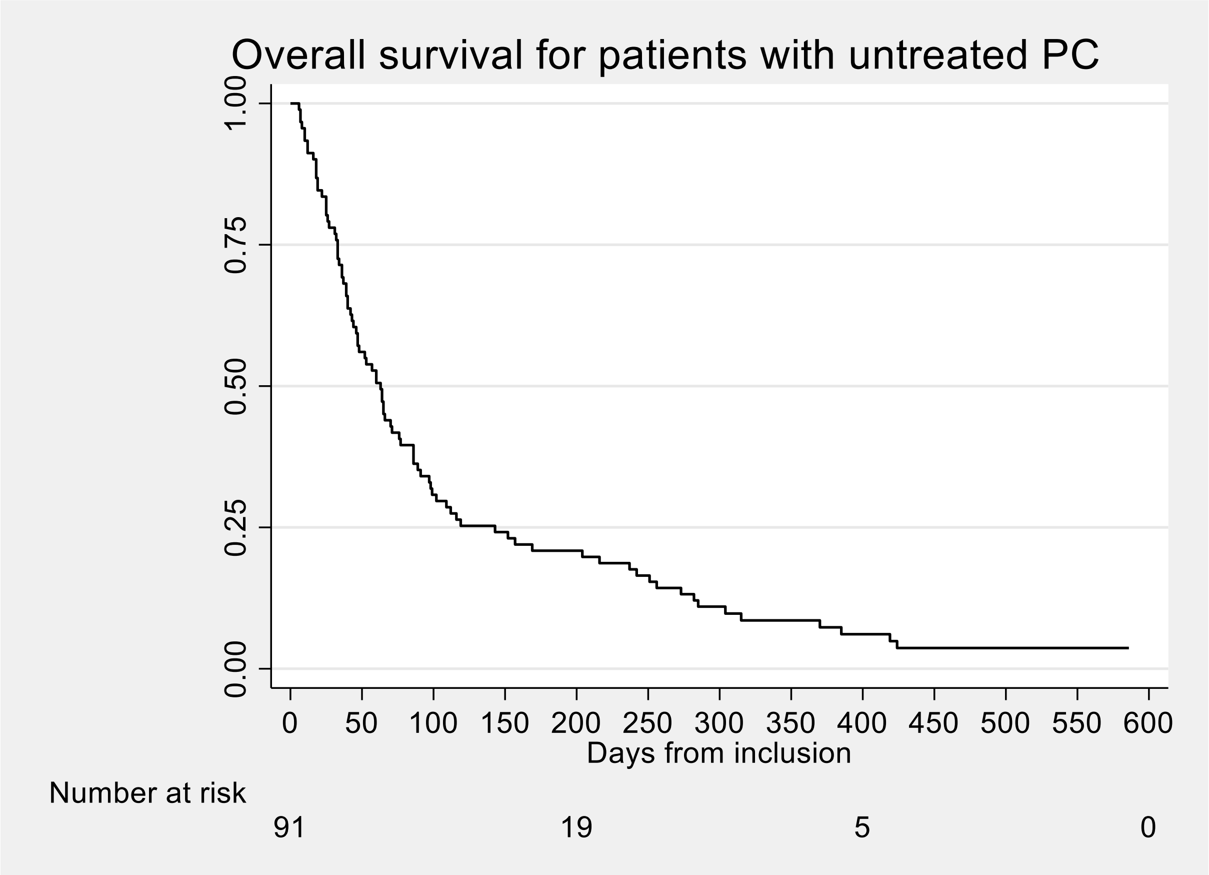

Figure S1. Kaplan-Meier plot of overall survival (OS) for 91 patients with untreated pancreatic cancer (PC) (median OS = 63 days).

Table S1. Reasons for opting out treatment in 91 patients with pancreatic cancer*.

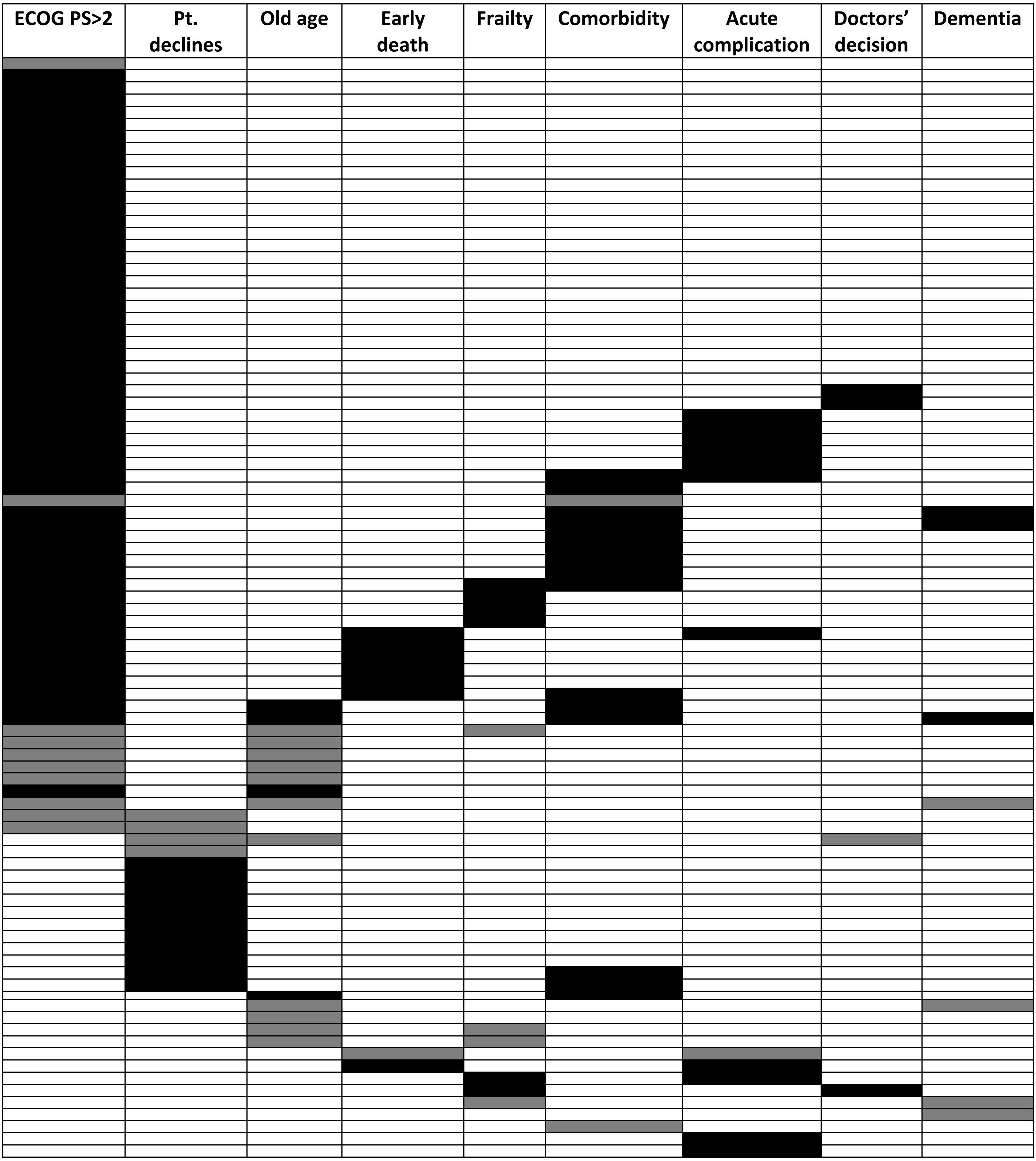

Note: ECOG PS, Eastern Cooperative Oncology Group performance status.

*Assessed by audit of electronic health records. Each row represents one patient. Gray squares, patients in stage I-II; black squares, patients in stage III-IV.
